# Junction of the redox dynamic, orchestra of signaling, and altered metabolism in regulation of T- cell lymphoma

**DOI:** 10.3389/fonc.2023.1108729

**Published:** 2023-05-19

**Authors:** Shantanu Singh, Akhilendra Kumar Maurya

**Affiliations:** Biochemistry and Molecular Biology Laboratory, Centre for Advanced Study in Zoology, Institute of Science, Banaras Hindu University, Varanasi, India

**Keywords:** T-cell lymphoma, redox status, cancer metabolism, Nrf2, NF-κB, signaling pathways

## Abstract

T-cell lymphoma is a hematologic neoplasm derived from the lymphoid lineage. It belongs to a diverse group of malignant disorders, mostly affecting the young population worldwide, that vary with respect to molecular features as well as genetic and clinical complexities. Cancer cells rewire the cellular metabolism, persuading it to meet new demands of growth and proliferation. Furthermore, the metabolic alterations and heterogeneity are aberrantly driven in cancer by a combination of genetic and non-genetic factors, including the tumor microenvironment. New insight into cancer metabolism highlights the importance of nutrient supply to tumor development and therapeutic responses. Importantly, oxidative stress due to an imbalance in the redox status of reactive species *via* exogenous and/or endogenous factors is closely related to multiple aspects of cancer. This alters the signaling pathways governed through the multiple intracellular signal transduction and transcription factors, leading to tumor progression. These oncogenic signaling molecules are regulated through different redox sensors, including nuclear factor-erythroid 2 related factor 2 (Nrf2), phase-II antioxidant enzyme, and NQO1 (NADPH quinone oxidoreductase (1). The existing understanding of the molecular mechanisms of T-cell lymphoma regulation through the cross-talk of redox sensors under the influence of metabolic vulnerability is not well explored. This review highlights the role of the redox dynamics, orchestra of signaling, and genetic regulation involved in T-cell lymphoma progression in addition to the challenges to their etiology, treatment, and clinical response in light of recent updates.

## Introduction

Genetic mutations, which are fuel for cancer, have been linked to different human malignancies. Every day, hundreds of cells undergo mutation in the human body, but they do not all go through tumor formation. However, under adverse conditions, adaptive changes may cause cells to behave abnormally, changing survival, growth, and division patterns, which potentially disturbs cellular and social control (1). The multiple rewirings of the cell’s metabolic, genetic, and signaling pathways resulting in a very disruptive destiny for the cell’s social environment. Lymphoid cells, a critical component of the immune system, have been identified as emerging players associated with the progression of multiple forms of cancer, including lymphoma. Lymphoma is a malignant disease consisting of a large heterogeneous group of lymphocytic cells that reside within the lymph nodes, spleen, thymus, bone marrow, and other parts of the body. Broadly, it is classified into Hodgkin’s and non-Hodgkin’s lymphoma (2). Hodgkin’s lymphoma is named after Dr. Thomas Hodgkin who discovered this disease in 1832. Hodgkin’s lymphoma is classified on the basis of the presence of a specific type of cell called a ‘Reed-Sternberg cell’, which is absent in its parallel subtype non-Hodgkin’s lymphoma (2). According to the ‘5th edition of WHO classification of lymphocyte neoplasm 2022’, tumor heterogeneity represents around 115 different types of lymphoid descendants (3). Non-Hodgkin’s lymphoma is classified as abnormal clonal proliferation of T cells and B cells that lack Reed-Sternberg cells, where the majority of them (80–90%) arise from B-type lymphocytes and the rest (10–20%) originate from T lymphocyte or natural killer cells (4). Lymphocytes have a definite life span; however, under certain circumstances they start dividing, causing them to aggregate in lymph nodes, which results in swelling of the lymphatic system. Various immune-suppressed pathologies, including HIV, receiving high doses of chemotherapy, organ transplantation, stem cell therapy, and even infection with Epstein bar virus and *Helicobactor pylori* bacteria, may cause a predisposition to non-Hodgkin’s lymphoma (5). This review focused on types and subtypes of lymphoma, specifically T-cell Lymphoma, and their dynamic regulation under the influence of redox status, metabolic tuning, and signaling pathways.

## T-cell lymphoma

The human body responds to foreign invaders with the help of lymphoid cells and the lymphoid system, including all cells of adaptive and innate immunity. T-cell lymphoma is hematologic neoplasm derived from the lymphoid lineage, which normally governs immune responses. Abnormality in this immune regulation has been reported in post-thymic or activated T lymphocytes. As per the report of the American Cancer Society, T-cell lymphoma accounts for about 10% of all lymphoma. WHO classified T-cell lymphoma into 39 types and 11 different categories (3). Different types of T-cell Lymphoma are listed in **Table 1**. Based on the involvement of bone marrow, it can be recognized as either lymphoma and/or acute lymphoblastic leukemia. It starts at the thymus site of T-cell maturation and develops into a tumor at the mediastinum and can spread to almost any part of the body, including the liver, bone marrow, gastrointestinal tract, spleen, skin, and brain (6).

**Table 1:** T-cell Lymphoma types.

SN		T-cell lymphoma	Subtypes
1		Tumor-like lesions with T-cell predominance	Kikuchi-Fujimoto disease
			Indolent T-lymphoblastic proliferation
			Autoimmune lympho-proliferative syndrome
2		Precursor T-cell neoplasm	T-lymphoblastic leukemia/lymphoma
			Early T-precursor lymphoblastic leukemia/lymphoma
3		Mature T-cell and NK-cell neoplasm	
	a	Mature T-cell and NK-cell leukemia	T-prolymphocytic leukemia
			Tlarge granular lymphocytic leukemia
			NK-large granular lymphocytic leukemia
			Adult T-cell leukemia/lymphoma
			Sezary syndrome
			Aggressive NK-cell leukemia
	b	Primary cutaneous T-cell lymphoma	Primary cutaneous CD4-positive small or medium T-cell lympho-proliferative disorder
			Primary cutaneous acral CD8-positive lympho-proliferative disorder
			Mycosis fungoides
			Primary cutaneous CD30-positive T-cell lympho-proliferative disorder: Lymphomatoid papulosis
			Primary cutaneous CD30-positive T-cell lympho-proliferative disorder: Primary cutaneous anaplastic large cell lymphoma
			Subcutaneous panniculitis-like T-cell lymphoma
			Primary cutaneous gamma/delta T-cell lymphoma
			Primary cutaneous CD8-positive aggressive epidermotropic cytotoxic T-cell lymphoma
			Primary cutaneous peripheral T-cell lymphoma
	c	Intestinal T-cell and NK-cell lymphoid proliferations and lymphoma	Indolent T-cell lymphoma of the gastrointestinal tract
			Indolent NK-cell lymphoproliferative disorder of the gastrointestinal tract
			Enteropathy associated T-cell lymphoma
			Monomorphic epitheliotropic intestinal T-cell lymphoma
			Intestinal T-cell lymphoma
	d	Hepatosplenic T-cell lymphoma	Hepatosplenic T-cell lymphoma
	e	Anaplastic large cell lymphoma	ALK-positive anaplastic large cell lymphoma
			ALK-negative anaplastic large cell lymphoma
			Breast implant-associated anaplastic large cell lymphoma
			Nodal T-follicular helper (TFH) cell lymphoma
	f	Peripheral T-cell lymphoma	Peripheral T-cell lymphoma
	g	EBV-positive NK/T-cell lymphoma	EBV-positive nodal T- and NK-cell lymphoma
	h	EBV positive T-cell and NK-cell lymphoma	Severe mosquito bite allergy
			Extranodal NK/T-cell lymphoma
			Hydroavacciniforme lympho-proliferative disorder
			Systemic chronic active EBV disease
			Systemic EBV-positive T-cell lymphoma of childhood

“a-h” represents subdivision of mature T-cell and NK-cell leukemia.

Epithelial–mesenchymal transition (EMT) is initiated in a malignant T-cell lymphoma, which allows epithelial cells to be converted into mesenchymal cells and moves them into distinct locations through a process known as metastasis. Chemotherapy is the most preferred treatment option, being preferable over radiation and surgery. However, a plethora of side effects, including chemotherapy-induced peripheral neuropathy (CIPN) and immunosuppression, can be seen in many parts of the body, including the brain, where it may display lifelong persistence (7–9). The antioxidant defense system is crucial in maintaining cellular homeostasis. An imbalanced redox status due to high metabolic demand and the microenvironment of T-cells could be targeted for treatment.

## Redox status and antioxidant defense system

Healthy cells routinely encounter immense amount of stress due to environmental (xenobiotic exposure, microbial exposure, etc.) and cellular (metabolic, immune function, etc.) responses in form of reactive oxygen species (ROS). These ROS are collectively defined under two subtypes, free radical species, including superoxide anions (O_2_^•−^), hydroxyl radicals (HO•), peroxyl (RO_2_•), and alkoxyl (RO•), and non-radical species, such as hydrogen peroxide (H_2_O_2_) (10). Accumulation of ROS cumulatively builds up in the cell in the form of oxidative stress. Oxidative stresses are imbalances between oxidants and antioxidants, leading to modification of biochemical properties of biomolecules in cells, which is balanced by an intracellular antioxidant defense system (11). Modulation of redox states contributed to multistage carcinogenesis either by a direct mechanism involving damage of DNA or indirectly by modulating cellular signal transduction (12, 13). ROS affect the function of multiple cellular proteins either by stabilizing them or modifying them in such a way that they can easily interact with their activator for their downstream target where they cause prolonged or constitutive activation of growth factors, cytokines, etc. NF-κB is a potent target of ROS that activates multiple transcription factors associated with cell cycle progression and survival (12). Elevated oxidative stress has been reported in many types of cancer cells where the redox changes have significant consequences. The application of antioxidants on T-cell lymphoma for therapeutic response has been extensively studied.

## Models for T-cell lymphoma study

For future drug development and etiological investigation there is a need for a suitable model that can mimic maximum possible properties of problems from the preclinical to clinical grade. A wide range of preclinical models are being studied in research ranging from primary cultures, cell lines, xenografting, and patient derived xenografting (PDX) to more advanced organoid cultures including the patient-derived organoid (PDO). Due to the diversity and/or heterogeneity found in T-cell malignancy a wide variety of *in vivo* as well as *in vitro* models have been established (13, 14). Various animal models used in T-cell lymphoma are listed in **Table 2**. Our elucidation of T cells in this review is mainly dominated by a transplantable T-cell lymphoma model also known as Dalton’s lymphoma. It has shown maximum association with a range of clinical markers of T-cell lymphoma. A wide range of markers, including regulation of CD3^+^, massive depletion of immature CD4^+^, CD8^+^ and mature CD4^+^, CD8^-^, and CD8^-^ along with impaired regulation of immunoregulatory cytokines such as IFN-ϒ, IL-10 and IL-2 for T-cell lymphoma, are listed in **Table 3**.

**Table 2:** T-cell lymphoma model.

SN	Type	Model	Mechanism	Effect	Reference
1	Angioimmunoblastic T-cell lymphoma	Roquin mouse model	A missense (M199R) San Roque mutation in the roquin gene	Increase of THF cell	(15)
		Tet2 gene trap mice model	A gene-trap vector inserted into the Tet2 section intron	THF- like phenotype	(16)
		G17V RHOA mouse model	By stabilizing G17V RHOA gene expression either retroviral transduction, knockout	Increases THF cell population	(17–19)
		PDX Models of angioimmunoblastic T-cell lymphoma	Inoculation of cells from lymph node of AITL to NOG mice	AITL-like disease	(20)
2	Anaplastic large T-cell lymphoma	NPM1-ALK transgenic models	By transplanting bone marrow cells transduced with a retroviral vector carrying NPM1-ALK cDNA into lethally irradiated mice and using transgenic approaches	Result in development of plasmacytoma, histiocytic malignancy, and large cell lymphoma from B-lineage	(21–26)
		PDX Models of anaplastic large-cell lymphomas	Inoculation of CD30+ALCL cell from patient to SCID mice	ALCL, ALK+ disease	(27)
3	Human T-cell lymphotropic virus type 1 adult T-cell leukemia/lymphoma	Mice expressing HTLV-1 viral proteins	Use of transgenics mice expressing Tax under the control of viral promoters HTLV1	Development of mesenchymal tumor in nose, ear, foot, and tail	(28)
		PDX Models of adult T-cell leukemia/lymphoma	Xenograft of patient derived cell to SCID and NOD/SCID mice	lymphocytic infiltration in spleen, liver, lung, and other organs	(29)
4	Cutaneous T-cell lymphoma	IL‐15 transgenic model	Transgenic mice model having increased IL-15 expression in CD4^+^T-cell	Fatal leukemia in skin	(30, 31)
		JAK3A572V mutant model	Retroviral induction of JAK3^A572V^ mutant cDNA into 5-flurouracil-treated murine bone marrow cells	CD8^+^ leukemic condition in the skin	(32, 33)
5	Enteropathy-associated T-cell lymphoma	Setd2cKOmicewere generated with Lck-Cre transgenic mice	Through transgenic and knockout processes	Increased no of ϒδ^+^T-cell in intraepithelial region	(34)

**Table 3:** T-cell lymphomas and their associated markers.

SN	T-cell neoplasm	Associated marker	References
1	T-prolymphocytic leukemia	Positive for CD3 and CD7 but negative for CD5 and CD30	(35–37)
2	T-large granular lympho-proliferative	Positive for CD3, CD7, and CD8 but negative for CD4, CD5, and CD30	(38–40)
3	Cutaneous ALCL (anaplastic large cell lymphoma)	Positive for CD3 and CD30 but negative for CD8	(40–42)
4	Hepatosplenic T-cell lymphoma	Positive for CD3 and CD7 but negative for CD4, CD5, CD8, and CD30	(38, 43, 44)
5	Angioimmunoblastic T-cell lymphoma	Positive for CD3 and CD5 but negative for CD7 and CD30	(38, 45)
6	Enteropathy associated T-cell lymphoma	Positive for CD3, CD5, and CD7	(46, 47)
7	Adult T-cell leukemia/lymphoma	Positive for CD3 and CD5 but not for CD7	(48, 49)

### Regulation of T-cell lymphoma by ROS

ROS governs multiple cellular responses and are collectively referred to as “redox messengers” (10). These messengers further initiate a cascade of events in a small cytoplasmic pool to regulate different cellular functional proteins. Here, we describe some of such signaling events that contribute to progression of T-cell lymphoma in response to these messengers while highlighting signaling molecules involved in it.

### Nrf2-NQO1 signaling

Nuclear factor erythroid 2-related factor 2 (Nrf2) is a leucine zipper transcription factor with 7-Neh (Nrf2 ECH homology) domains, each with a different function. Nrf2 is found in every cell type with basal level of activation and its level is regulated by 26s proteasomal degradation mediated by E3 ubiquitin ligase (Keap1-Cul3-Rbx) (50). There are around 200 genes with ARE (antioxidant response element) sequences which are regulated by Nrf2 (51, 52).

Cells are armored with intricate defense system to counter oxidative stress through the Keap1-Nrf2-ARE pathway (53). The Nrf2 protein is reported to be upregulated during oxidative stress, and knockout of Nrf2 results in severe deficiency in the coordination of the gene regulatory program with increased susceptibility to oxidative damage (54). Nrf2 is reported to be a double-edged sword with a dual role in controlling cancer (55). The role of Nrf2 in cancer is a little paradoxical, however, in T-cell lymphoma it is delimited to its traditional pathway where any deterioration in this pathway leads to carcinogenesis and restabilizing it results in the enhancement of lymphoma. Multiple drivers have been reported to activate or stabilize the expression of Nrf2. PKC (Protein kinase C) is one such sensor that stabilizes the translocation of Nrf2 from the cytosol to nucleus by phosphorylating it at the ser-40 position and facilitating its release from its cytosolic anchor Keap1 (56). Nrf2 protects the cell from oxidative damage by preventing cellular damage and halting cell cycle progression. Overexpression of PKC promotes tumor progression (57). Other accessory proteins such as ERK2 and GSK3β have also been reported to regulate Nrf2 activity (58–66).

**Figure 1:**
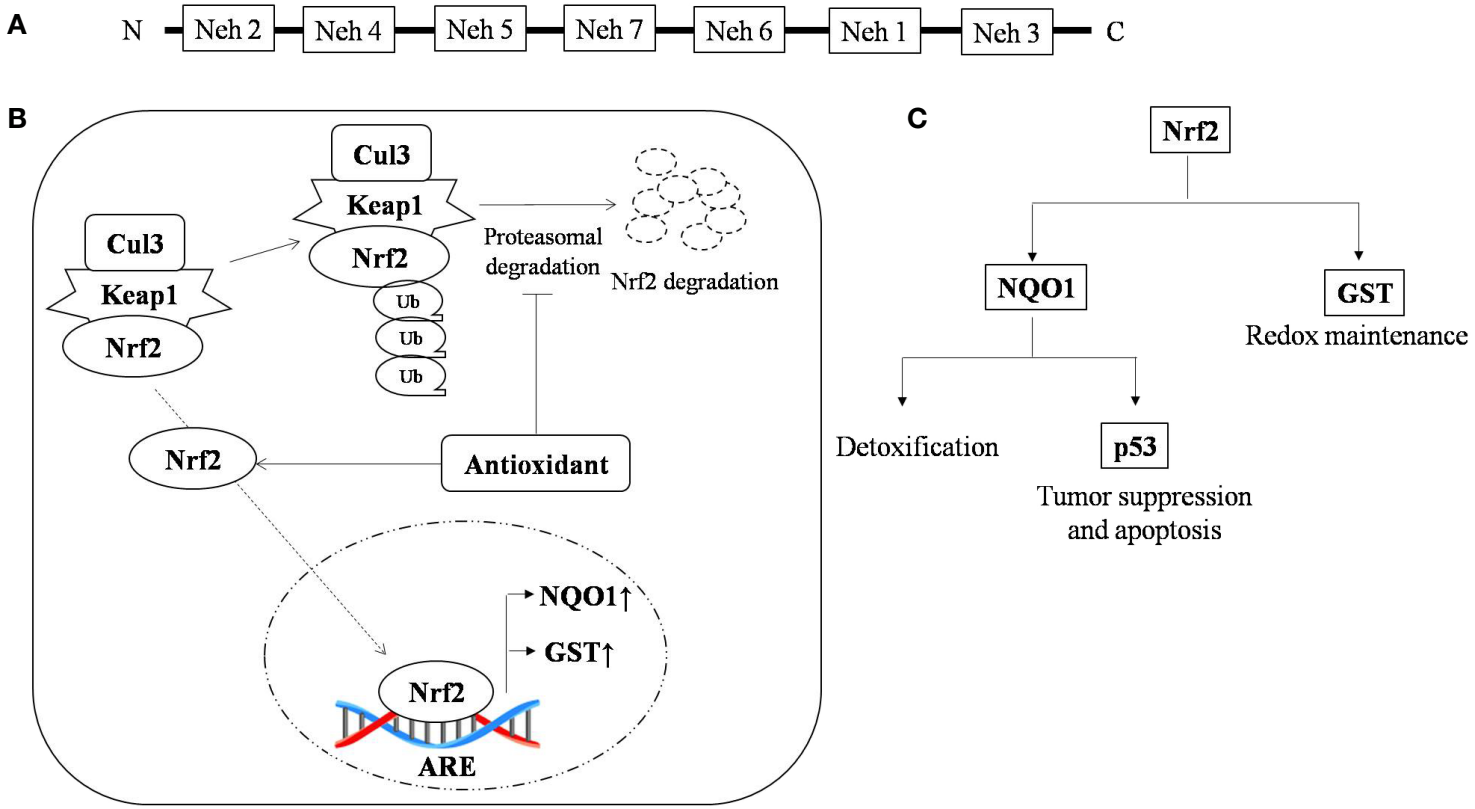
Nrf2 activation and the role of antioxidants in tumor suppression **(A)** Typical representation of the Nrf2 domain that regulate specific ARE genes; **(B)** Antioxidant stabilizes Nrf-2 by interfering in its proteasomal degradation pathway, resulting in enhanced binding of Nrf-2 with its promoter sequence, which causes upregulation of antioxidant scavengers NQO1 and GST and **(C)** Nrf-2 promotes the expression of the antioxidant scavengers, namely NQO1 and GST, where GST is a thiol compound regulating redox status and NQO1 promotes detoxification and expression of p53, which under stress regulates cell cycle and apoptosis. ↑, up-regulation; ↓, down-regulation.

Genomic response by antioxidant signaling inducers suggest NAD(P)H:quinone oxidoreductase 1 (NQO1) and glutathione S-transferase (GST) are the two main target genes activated through Nrf2 in T-cell lymphoma (67, 68). NQO1 is a phase two antioxidant enzymes; it catalyzes the reduction of several environmental electrophonic contaminants and endogenous compounds, including quinones and nitro compounds (69). NQO1 under the regulation of Nrf2 plays a dual role in protection against carcinogenesis. Nrf2-dependent induction of NQO1 downregulated lipopolysaccharide (LPS)-induced expression of inflammatory cytokines, thereby impairing the inflammatory responses (70). It has also reported to induce apoptosis through p53. p53 is a tumor suppressor protein transcribed in response to cellular alteration like DNA damage and accumulates inside the cell to limit cell proliferation by initiating cell cycle arrest and apoptosis (71). Glutathione s-transferase (GST) is the second important target of the antioxidant-induced downstream response of Nrf2, which regulates metabolic detoxification of various electrophilic xenobiotic by forming a complex with them. Several isoforms of GST are reported to be expressed in a tissue-specific manner (72). The GST null phenotype is associated with an increased rate of cancer development (73). Nrf2 activation and its role in tumor suppression is depicted in **Figure 1**. Curcumin, an antioxidant, has shown anti-carcinogenic properties by elevating GSTα, GSTµ, and GSTπ activity (74).

### NF-κB signaling

**Figure 2:**
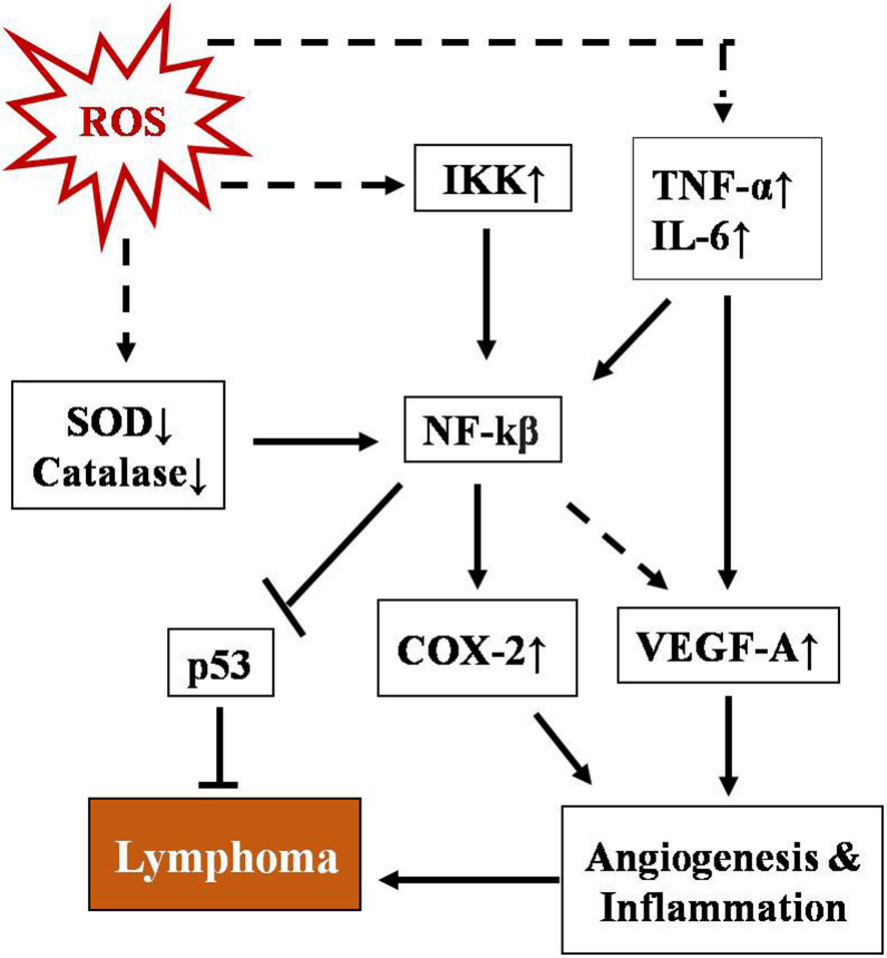
Schematic representation of regulation of T-cell lymphoma by NF-κB. NF-kB activation involves a phosphorylation cascade leading to degradation of its inhibitor IKβ. ROS regulate NF-κB through multiple cellular agents, namely SOD↓, catalase↓, Ikk↑, TNFα, and IL-6↑ (where ↑& ↓represent upregulation and downregulation respectively). Activated NF-κB leads to upregulation of COX-2 and VEGF-A and downregulates p53, promoting angiogenesis and inflammation which ultimately lead to lymphoma.

NF-κB represents a group of proteins that are very sensitive to any changes in the cellular redox profile and has a key role in cancer progression (75, 76). Normally, it remains sequestered in the cytoplasm along with inhibitory subunit Ikβ. ROS have been reported as both activators and inhibitory stimuli for NF-κB depending on the type of modification and site of action (77–80). NF-kB regulates multiple proteins, including COX2, VEGE-A, TNF-α, and IL-6. The COX2 protein has been described as one of the major players linking inflammation to cancer through different signaling pathways (81, 82). Vascular endothelial growth factor-A (VEGF-A), another positive downstream of NF-κB, promotes angiogenesis by activating its receptor VEGF-R1 in endothelial cells of micro vessels in lymphoma (83). A schematic representation of the regulation of T-cell lymphoma by NF-κB is depicted in **Figure 2**. NF-κB has also been found to regulate inflammatory cytokines in a positive feedback mechanism such as TNF-α and IL-6 in T-cell malignant transformation (84).

### PI3K-AKT signaling

**Figure 3:**
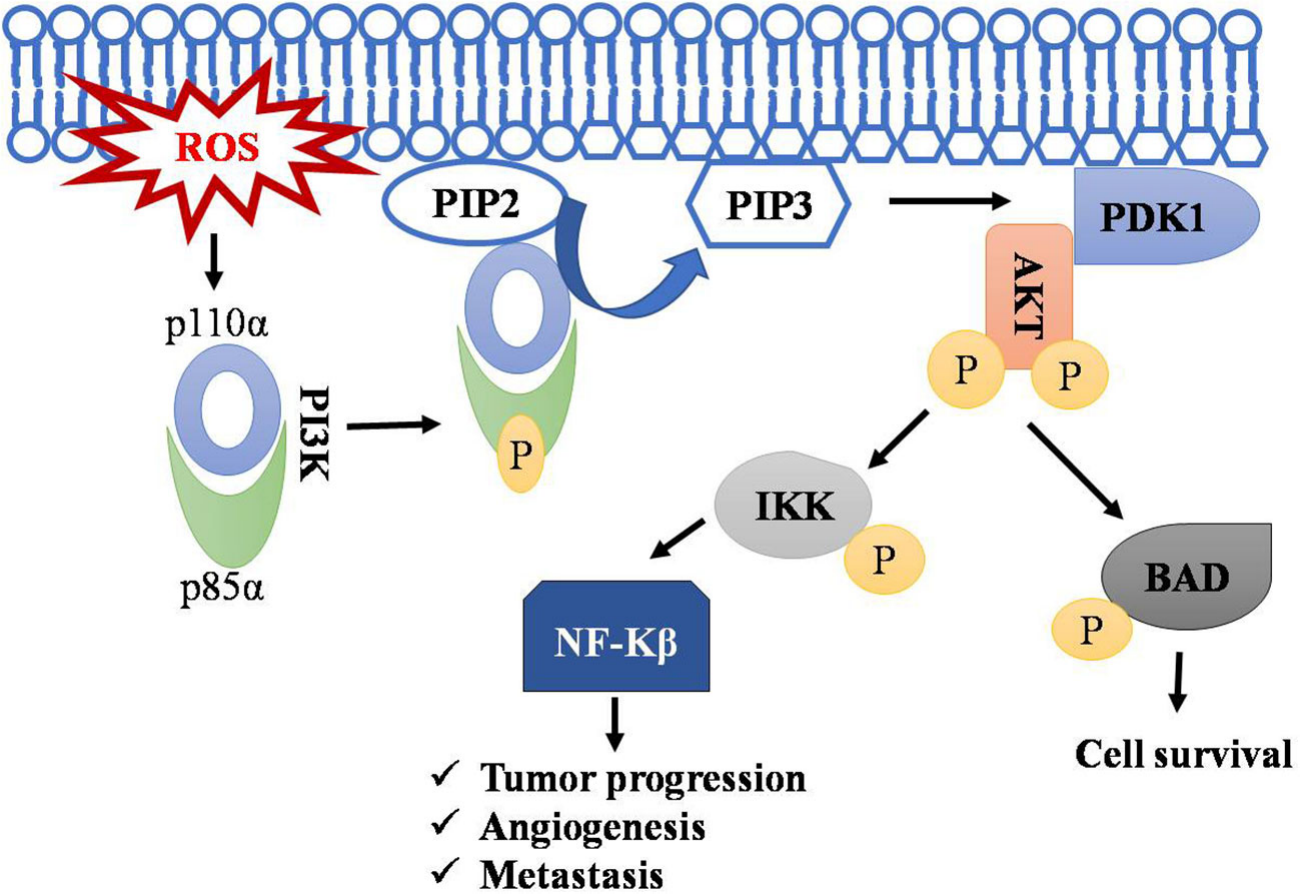
Regulation of T-cell lymphoma by PI3k-AKT pathway. Accumulation of ROS leads to the phosphorylation of PI3K resulting in disassembly of its regulatory subunit (p85α) and catalytic subunit (p110α). The catalytic subunit now phosphorylates PIP2 into PIP3. PIP3 serve as a docking site for AKT, where PDK1 activates AKT by phosphorylation. AKT phosphorylates IKK and BAD, leading to NF-kB activation and thus to cell survival, angiogenesis, and metastasis. Symbol "✔" represents just a separate process. Arrow represents the sequential progression of signaling.

PI3K signaling acts as master regulator in controlling cell survival, proliferation, and growth. It catalyzes the conversion of phosphatidyl inositol (4, 5)-bisphosphate (PIP_2_) to phosphatidylinositol (3–5) - triphosphate (PIP_3_), and this PIP3 initiates the localization of AKT to membrane and its subsequent activation by PDK1.ROS accumulation leads to phosphorylation of PI3K on tyrosine residues of a regulatory subunit (p85α) and thereby results in disassembly of it from its catalytic counterpart p110α (85–87). The p110α triggers rapid transformation of PIP2 to PIP3. AKT activation resulting in cell transformation and generation of cancer phenotypes (88). AKT promotes cell survival by inhibiting proapoptotic protein BAD by phosphorylating it and preventing its interaction with BCL-xl (85, 89–91). Regulation of T-cell lymphoma by the PI3k-AKT signaling pathway is depicted in **Figure 3**.

## Conclusion and future perspective

From increasing experimental evidence, the role of ROS in tumorigenesis and its progression by regulating and/or altering the cellular environment has become apparent, stabilizing it as a potent hallmark of cancer. Although we have discussed some of the pathway, their complete regulation machinery is unknown and needs more scientific exploration. Changes in cellular redox are stabilized to contribute toward cancer pathogenesis but specific redox levels and their origin are still matters of research. Associated signaling pathways could reveal the hurdle of tumor progression by providing a target for drug development and help us better understand the etiology of cancer progression.

## Author contributions

S.S.: writing-original draft and writing- review and editing. A.K.M.: conception and design, writing-original draft, writing-review and editing, administrative, technical, and material support. All authors listed have made a substantial, direct, and intellectual contribution to the work, and approved it for publication.
